# ROS and *Trypanosoma cruzi*: Fuel to infection, poison to the heart

**DOI:** 10.1371/journal.ppat.1006928

**Published:** 2018-04-19

**Authors:** Claudia N. Paiva, Emiliano Medei, Marcelo T. Bozza

**Affiliations:** 1 Departamento de Imunologia, Instituto de Microbiologia, Universidade Federal do Rio de Janeiro (UFRJ), Rio de Janeiro, Brazil; 2 Instituto de Biofísica Carlos Chagas Filho, Universidade Federal do Rio de Janeiro, Rio de Janeiro, Brazil; 3 Centro Nacional de Biologia Estrutural e Bioimagem, Universidade Federal do Rio de Janeiro, Rio de Janeiro, Brazil; Boston College, UNITED STATES

## Abstract

The activation of macrophage respiratory burst in response to infection with *Trypanosoma cruzi* inflicts oxidative damage to the host’s tissues. For decades, the role of reactive oxygen species (ROS) in the elimination of *T*. *cruzi* was taken for granted, but recent evidence suggests parasite growth is stimulated in oxidative environments. It is still a matter of debate whether indeed oxidative environments provide ideal conditions (e.g., iron availability in macrophages) for *T*. *cruzi* growth and whether indeed ROS signals directly to stimulate growth. Nitric oxide (NO) and ROS combine to form peroxynitrite, participating in the killing of phagocytosed parasites by activated macrophages. In response to infection, mitochondrial ROS are produced by cardiomyocytes. They contribute to oxidative damage that persists at the chronic stage of infection and is involved in functional impairment of the heart. In this review, we discuss how oxidative stress helps parasite growth during the acute stage and how it participates in the development of cardiomyopathy at the chronic stage.

## Shifting the old paradigm

It was back in 1968 that the production of reactive oxygen species (ROS) by phagocytes during respiratory burst was proposed as a killing mechanism of pathogens [1]. The respiratory burst of phagocytes quickly became the most well-studied mechanism of microbial killing, and by the late 1970s to the early 1980s, *T*. *cruzi* was assumed to be killed by ROS from macrophages [2–5], despite contradictory evidence [6–9] that went unnoticed for a decade.

In 2012, *T*. *cruzi* was found to grow under oxidative conditions both in macrophages and in vivo, and antioxidants were detrimental to its growth [10]. Since then, several publications have followed this idea. Apparently, the killing of *T*. *cruzi* by ROS from respiratory burst was established as a paradigm in the literature on weak grounds [6, 7, 10]. Here, we bring together important works approaching how ROS production by the host promotes infection and how it contributes to Chagas heart disease at the chronic stage.

A confounding factor in the study of the relationship between ROS and *T*. *cruzi* is the finding of trypanocidal drugs that cause oxidative stress. The trypanocidal activity of benznidazole, for instance, was credited to benznidazole’s capacity to cause oxidative damage to the parasite [11]. However, benznidazole failed to generate ROS in the presence of *T*. *cruzi* extracts in vitro [12]. Accordingly, benznidazole’s trypanocidal actions were assigned to the reduction of its nitro group by trypanosomal type I nitroreductase [13], followed by the covalent linking of reduced benznidazole to internal thiols (e.g., trypanothione) [14] and generation of DNA-toxic glyoxal adducts [15] in an oxygen-insensitive reaction. Although the full mechanism of action of benznidazole remains to be elucidated, these results indicate that ROS are not involved in benznidazole’s trypanocidal effects. Benznidazole also activates nuclear erythroid factor-2 (Nrf2) and protects the host from oxidative damage in a delayed time frame [16].

What also has been found confusing is the decreased infectivity found in parasites unable to degrade ROS or repair oxidative damage [17–19]. Unguarded parasites succumb to oxidative damage instead of harvesting the potential benefits of living in an oxidative environment, such as the availability of iron found inside macrophages under oxidative stress, or the proliferation in response to ROS signaling. The antioxidant enzymes expressed by *T*. *cruzi* are crucial to defending against oxidative damage, allowing the parasite to thrive in oxidative conditions [20–22].

## Evidences in favor of ROS as a promoter of *T*. *cruzi* infection

Increased production of ROS and the resulting oxidative damage have been largely documented during both acute and chronic stages of *T*. *cruzi* infection with strains from different discrete typing units (DTUs) [23–26], and have been ascribed to NADPH oxidase 2 (NOX2) activation in unstimulated infected macrophages [25, 27]. Thus, protection against oxidative stress has the potential to reduce tissue damage in Chagas disease.

Since even low numbers of parasites cause progressive tissue damage, inducing disease tolerance against oxidative damage was tested as a useful strategy to treat Chagas disease [28, 29]. Since the beginning of infection, mice were treated with cobalt protoporphyrin (CoPP), an antioxidant that induces disease tolerance to malaria mostly by inducing heme oxygenase (HO-1) expression through Nrf2 activation [27, 30]. Even though we expected antioxidants to increase parasite burden, they did not: CoPP actually reduced parasite burden. Parasitemia and macrophages, heart, and skeletal muscle parasitism all reacted similarly to CoPP. No known effects of HO-1 induction [31–33] could explain this reduction of parasitism. What is more, no evidence was found that CoPP affected trypomastigote viability [27].

**Fig 1:**
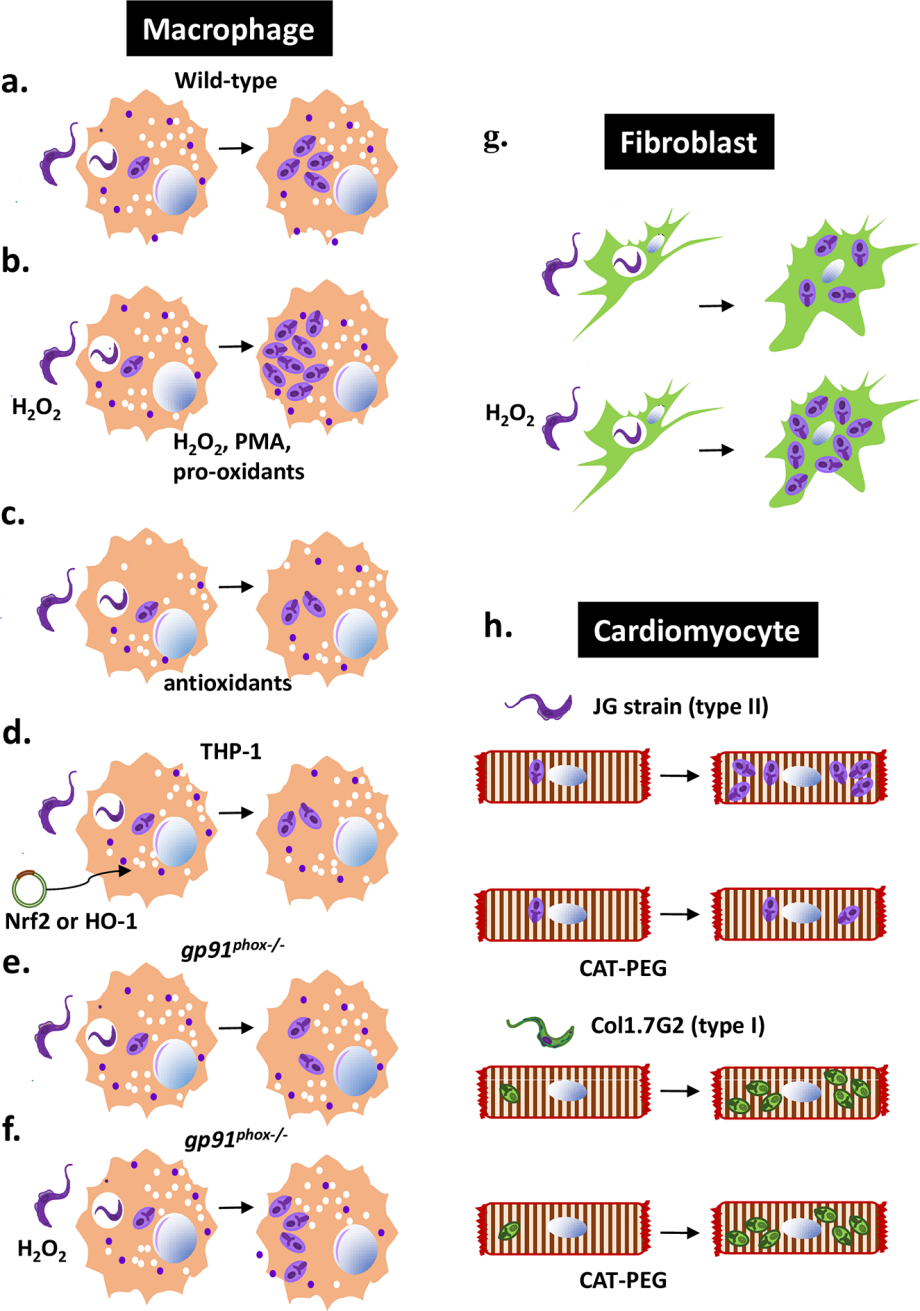
Oxidative stress fuels *T*. *cruzi* infection in macrophages, fibroblasts, and cardiomyocytes. (a) Parasite burden of nonactivated C57BL/6 macrophages infected with CL-Brener (type VI) or Y strain (type II) trypomastigotes and cultivated in medium for 48 h. (b) Infected macrophages incubated with H_2_O_2_, PMA, or pro-oxidants have increased parasite burden [17, 27]. A similar result is obtained if parasites are treated with H_2_O_2_ before infection [17]. (c) Infected macrophages incubated with antioxidants have decreased parasite burden. (d) THP-1 human macrophage lineage transfected with an Nrf2- or HO-1-containing plasmid before infection presents smaller parasite burden than cells transfected with an empty expression plasmid [27]. (e) Infected *gp91*^*phox-/-*^ macrophages have smaller parasite burden than wild-type macrophages [17, 27]. (f) Trypomastigotes treated with H_2_O_2_ before infecting *gp91*^*phox-/-*^ macrophages give rise to parasite burden similar to that of nontreated trypomastigotes growing in wild-type macrophages, as in (a) [17]. (g) Trypomastigotes treated with H_2_O_2_ before infecting fibroblasts also give rise to increased parasite burden [50]. (h) Cardiomyocytes infected with the JG strain (type II) respond to CAT-PEG with decreased burden compared to nontreated cells, while cardiomyocytes infected with Col1.7G2 (type I) are unresponsive to CAT-PEG [57]. CAT-PEG, catalase conjugated to polyethylene glycol to permeate the cells; HO-1, heme oxygenase; JG, type II T. cruzi strain; Nrf2, nuclear erythroid factor-2; PMA, phorbol 12-myristate 13-acetate.

The first study launched to systematically assess the effects of oxidative stress on the parasite burden during acute *T*. *cruzi* infection contradicted the paradigm of trypanocidal ROS. Treatment with CoPP reduces the parasite burden of infected macrophages from HO-1-knockout but not from Nrf2-knockout mice, while transfection of THP-1 macrophages with either Nrf2 or HO-1 reduces the parasite burden (Fig 1D); these two observations indicate that the effects of CoPP depend on the redundant action of Nrf2-controlled genes [27]. Incubation of infected macrophages with several antioxidants (NOX2-inhibitor apocynin, glutathione-replenisher N-acetyl-L-cysteine [NAC], polyphenols resveratrol and pterostilbene, biliverdin, bilirubin, catalase-polyethylene glycol [PEG], or superoxide dismutase [SOD]-PEG) reduces parasite burden, while respiratory burst–inducer phorbol 12-myristate 13-acetate (PMA), pro-oxidant paraquat, and H_2_O_2_ increase it (Fig 1B and 1C). Macrophages taken from *gp91*^*phox-/-*^ (which lacks NOX2) and infected in vitro have smaller parasitism than their wild-type counterparts [17, 27]. When *gp91*^*phox-/—*^infected macrophages are incubated with H_2_O_2_, the infection index increases [17], indicating that H_2_O_2_ supplies an additional proliferative stimulus missing in *gp91*^*phox-/-*^ macrophages (Fig 1E and 1F). Peritoneal macrophages taken from in vivo infected *gp91*^*phox-/-*^ mice have decreased parasite burden, and parasitemia was moderately decreased [27]. This latter result, however, was not confirmed in another work [17]. A general picture that emerges from these studies shows that ROS fuel *T*. *cruzi* infection in macrophages during the early acute stage. In fact, this was not the first work to show that ROS could favor an infection; they do fuel also other types of infection [10].

A question still to be answered is whether ROS can reach high concentrations inside invaded macrophages capable of killing or restraining amastigote growth instead of stimulating their proliferation. Although trypomastigotes escape the phagosome, H_2_O_2_ can diffuse through the cytoplasm and reach low micromolar concentrations [34]. The incubation of infected macrophages with up to 100 µM H_2_O_2_ promotes a more intensive amastigote proliferation, but the concentrations of H_2_O_2_ that reach the cytosol are unknown. Taking the cytosolic concentration of H_2_O_2_ during macrophage respiratory burst as a proxy, it would be within the 1–4 µM range [35, 36]. In fact, when macrophages infected with *T*. *cruzi* are activated with PMA, they give rise to a greater parasite burden [27], indicating that the concentrations reached during the respiratory burst are stimulatory. When trypomastigotes are directly incubated with H_2_O_2_ before invading gp91^phox-/-^ macrophages, they give rise to an increased amastigote burden at 100 µM, which demonstrates that trypomastigotes not only resist direct exposure to high H_2_O_2_ concentrations but also differentiate into amastigotes that preserve the memory of this encounter and grow more intensively [17]. However, incubation with H_2_O_2_ at 300 µM results in reduced parasite burden. Although no data on the effects of this concentration of H_2_O_2_ on macrophage invasion or parasite differentiation are available [17], we believe that this result is due to parasite elimination by direct oxidative damage. Nevertheless, a concentration of H_2_O_2_ as high as 300 µM is unlikely to occur in vivo [37], which suggests that the lethal oxidative damage of wild-type *T*. *cruzi* does not occur in physiological conditions.

In contrast to the early acute phase, by the end of the acute phase, splenic parasitism of *gp91*^*phox-/-*^ mice infected with Y strain increased [38]. Unlike C57BL/6 wild-type controls, *gp91*^*phox-/-*^ mice did not survive the infection [27, 38]. Since the extension of the treatment with the antioxidant CoPP until the end of the acute phase was counterproductive to the control of parasite burden and killed wild-type mice [27], we speculate that later, antioxidant therapy might impair the establishment of adaptive immunity. A general impairment of lymphocyte proliferation seems to accompany all ROS-deficient conditions in acute Chagas disease [24, 25, 27], and the establishment of adaptive immunity is fundamental to control parasitemia. The role played by ROS-producing macrophages at the chronic stage of infection has not been evaluated, but it is likely that most of them are activated by interferon gamma (IFNγ) and producing trypanocidal nitric oxide (NO) and peroxynitrite.

The idea that oxidative stress promotes parasitism of *T*. *cruzi* may help to explain some results reported in the literature. Cruzipain is an enzyme that increases the susceptibility of macrophages to *T*. *cruzi* infection [39] and is a major inducer of NOX2 activation during macrophage infection [40]. The genetic absence of signaling lymphocytic activation molecule family member 1 (*Slamf1*), a condition that reduces NOX2 activation in myeloid cells, was found to increase resistance to *T*. *cruzi* infection [41]. Aryl hydrocarbon receptor (*Ahr*) is a gene known to promote ROS production in response to infections such as listeriosis [42]; its genetic absence decreases *T*. *cruzi* parasite burden as well as ROS production in infected macrophages [43]. Also, the treatment of infected ES mice with melatonin [44] or curcumin [45], both known to activate antioxidant defenses, reduced parasite burden in blood and heart tissue. These results are all in agreement with the general notion that ROS promotes *T*. *cruzi* infection, as suggested many years ago [6–9].

## Mechanisms underlying *T*. *cruzi* growth in oxidative environment

**Fig 2:**
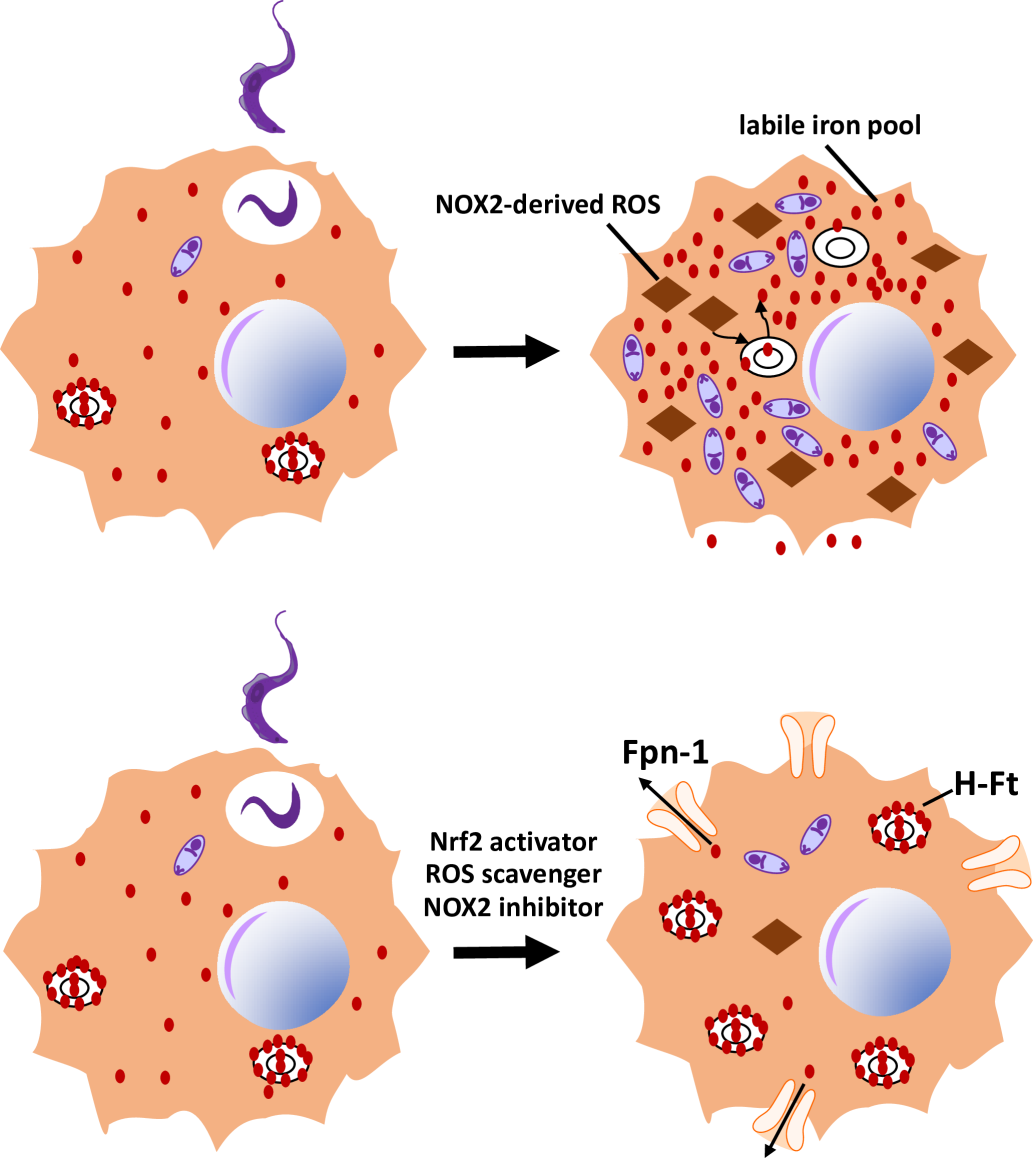
LIP is associated with oxidative conditions and parasite growth in macrophages. Treatment of infected macrophages (Y strain) with antioxidants (NAC, apocynin, CoPP) reduces ROS content, LIP, and parasite burden, while reducing the expression of H-Ft and increasing the expression of the iron channel Fpn-1 [27]. CoPP, cobalt protoporphyrin; Fpn-1, ferroportin-1; H-Ft, H-ferritin; LIP, labile iron pool; NAC, N-acetyl-L-cysteine; ROS, reactive oxygen species.

The mechanisms by which ROS promote *T*. *cruzi* infection are still to be fully learned. Antioxidants up-regulated H-ferritin (H-Ft, a cytosolic protein that binds iron) and ferroportin-1 (Fpn-1, a channel that allows iron efflux) expression, decreasing labile iron pool (LIP) in macrophages (Fig 2). Increasing LIP by several means resulted in increased parasite burden, while reducing LIP had the opposite effect. Iron also increased the parasite burden of infected *gp91*^*phox*-/-^ macrophages to an extent similar to that found in wild-type mice. These results point to the regulation of LIP as the mechanism underlying the increase in macrophage parasitism produced by oxidative stress. However, the research did not study whether the manipulation of LIP could work by interfering with oxidative stress itself, thereby stimulating *T*. *cruzi* proliferation.

Several research groups have explored the possibility that the oxidative environment is itself a direct stimulus to the growth of *T*. *cruzi*. Parasites proliferated in response to H_2_O_2_ [46]. The increased growth of epimastigotes from type I Dm28c clone in response to H_2_O_2_ involved a calmodulin-dependent protein kinase II (CAMKII)–like enzyme in the pathway to proliferation [47], and the mitochondrial ROS resulting from heme metabolism enhanced the growth [48]. The exposure of epimastigotes to different reduction–oxidation (redox) state environments was evaluated in vitro and in the gut of the insect vector: Antioxidants reduced proliferation and increased metacyclogenesis, while pro-oxidants had the opposite effect [49]. Trypomastigotes responded to incubation with H_2_O_2_ before the infection, giving rise to greater amastigote burden after they invaded *gp91*^*phox-/-*^ macrophages (Fig 1F) or fibroblasts [50] (Fig 1G). Altogether, these results indicate that ROS directly affect parasites.

Resistance to oxidative stress confers an advantage to parasites. While the incubation of infected macrophages with catalase-PEG reduces parasitism [17, 18, 27], parasites expressing catalase give rise to a far greater parasite burden in macrophages and in mice than wild-type parasites [18]. Parasites that overexpress an enzyme that hydrolyzes 8-oxo-dGTP (MutT) are more capable of withstanding oxidative damage to their DNA, presenting both an increased growth in macrophages, fibroblasts, and increased overall parasite burden in mice [17, 18, 50], as well as responding with an increased growth to H_2_O_2_ exposure prior to invading fibroblasts [50]. The incubation with catalase-PEG reduces the growth of these parasites in macrophages more than of wild-type parasites [18]. These results reinforce the idea that the better armed against oxidative stress *T*. *cruzi* parasites are, the more they reduce their growth in response to a less oxidative environment [50].

We speculate that two possible evolutionary pressures that forced the selection of *T*. *cruzi* proliferative response were the increased availability of nutrients (such as labile iron) and the decreased activation of efficient trypanocidal mechanisms under oxidative stress; these pressures could have possibly interacted. Also, we emphasize that ROS affect *T*. *cruzi* growth inside only unstimulated macrophages and thus are more likely to be relevant at the very beginning of infection, when the production of IFNγ is still low and most macrophages are not activated to produce NO.

## The case for peroxynitrite as a main trypanocidal species in activated macrophages

**Fig 3:**
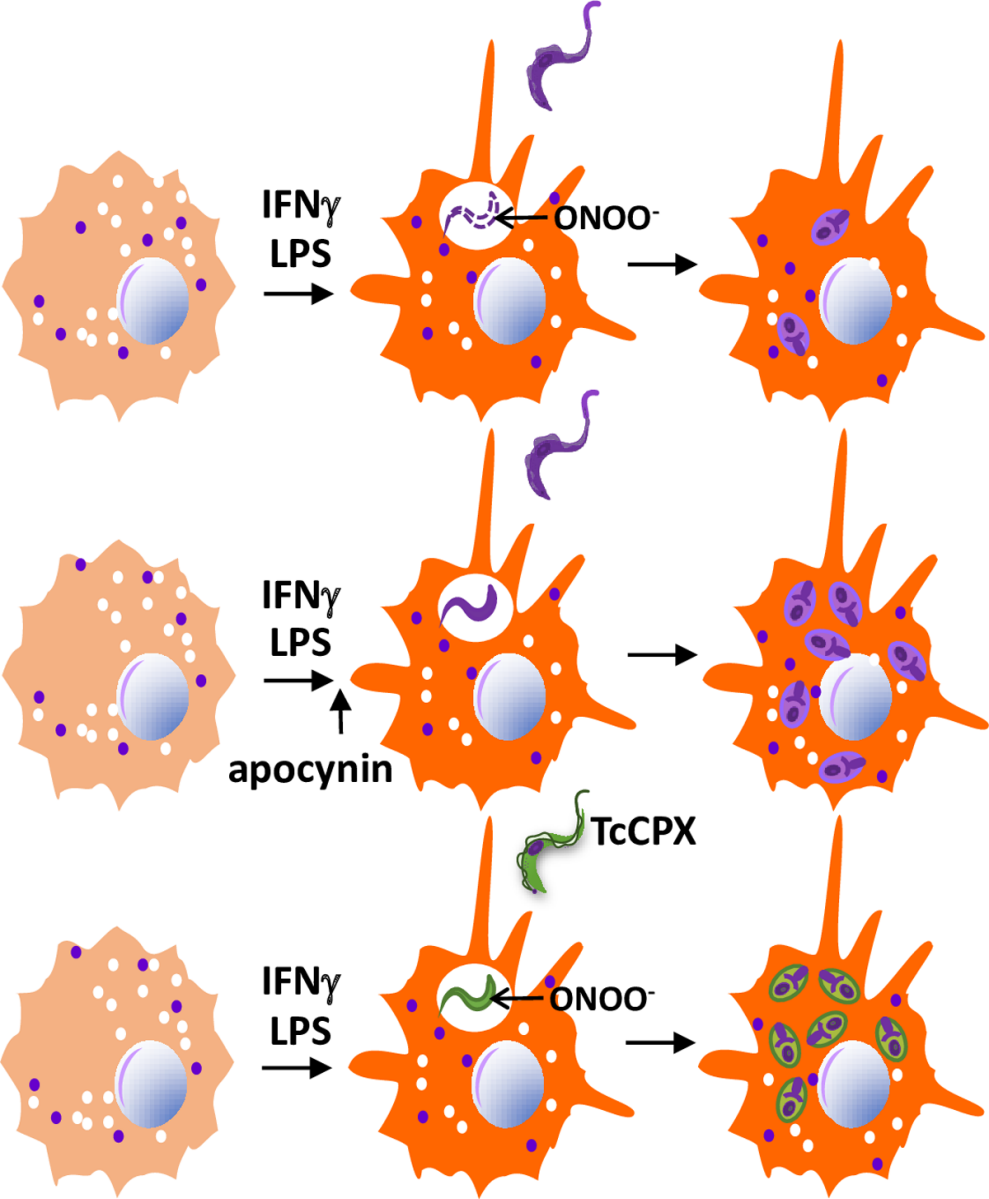
In macrophages activated before infection, ROS is detrimental to *T*. *cruzi* infection. Macrophages activated with LPS and IFNγ before infection eliminate phagocytosed *T*. *cruzi* CL-Brener by producing peroxynitrite. When the production of peroxynitrite is impaired by inhibiting NOX2-derived ROS with apocynin (right before the infection or after) or when *T*. *cruzi* expresses TcCPX, an enzyme that degrades peroxynitrite, the resulting parasite burden is increased [52]. IFNγ, interferon gamma; LPS, lipopolysaccharide; ROS, reactive oxygen species; TcCPX, triparedoxin peroxidase.

Peroxynitrite is a highly lethal weapon used by phagocytes against pathogens. It is formed when there is simultaneous production of NO and ROS [10]. When infected with *T*. *cruzi*, unstimulated macrophages produce little NO [7, 17, 51]. Radi and colleagues showed that in unstimulated, CL-Brener-infected macrophages, parasites remained well-preserved, although nitroblue tetrazolium was reduced, and superoxide and H_2_O_2_ predominated within the phagosomes [52]. They detected high amounts of peroxynitrite only when macrophages were infected after activation with lipopolysaccharide (LPS)/IFNγ, indicating that for these species to be present, additional factors must send a signal for NO production. Inside the peroxynitrite-producing macrophages, the authors observed peroxynitrite-dependent protein oxidation and damaged parasites (Fig 3).

Alvarez et al. also used a modified parasite that overexpressed a peroxynitrite-degrading enzyme to infect unstimulated macrophages, triparedoxin peroxidase (TcCPX). It grew similarly to wild-type parasites [52]. However, it produced an enhanced infection in IFNγ-activated macrophages and in both heart parasitism and the peak of parasitemia in mice, suggesting that when the immune system is activated in vivo, peroxynitrite production is probably an important mechanism of parasite killing.

In general, simultaneous ROS and NO production is associated with *T*. *cruzi* killing inside activated macrophages [53]. The differences in the role of ROS between unstimulated and activated macrophages mean that the activation of a naïve invaded macrophage is different from the active phagocytosis of *T*. *cruzi*. We assume that in non-redox-manipulated macrophages, peroxynitrite is the most likely trypanocidal species.

## Counterevidence for oxidative stress as a promoter of *T*. *cruzi* infection

During the course of acute infection with clone Sylvio X10/4, treatment with NOX-inhibitor apocynin increased parasite burden in blood, skeletal muscles, and hearts of mice [24]. Similarly, in the genetic absence of p47^phox^, a subunit responsible for NOX2 translocation to the membrane upon activation, Sylvio X10/4 parasites infected a higher percentage of bone marrow macrophages, besides leading to greater parasitemia and heart parasitism of mice [25]. These results greatly differ from those in which peritoneal macrophage parasitism decreased [27] and splenic parasitism decreased [17] in *gp91*^*phox-/-*^ (NOX2-deficient) mice infected with either Y strain and with clone CL-Brener. It is still unclear whether the differences are due to the distinct functions of these NOX2-subunits or to different responses of *T*. *cruzi* DTU types.

The association between high levels of ROS and reduced parasite burden in infected macrophages from *Nlrp3*^*-/-*^ mice was interpreted as an evidence for the contribution of ROS to *T*. *cruzi* (Sylvio X10/4) killing [54]. Other authors found that ROS were not involved in the control of interleukin 1 beta (IL-1β) production and that the genetic absence of *Nlrp3*^*-/-*^ in macrophages infected with Y strain increased parasitism [55]. The apparent reduction of parasite burden in THP-1 macrophages infected with Sylvio X10/4 and incubated with antioxidants apocynin and NAC seems to argue against the idea that ROS are involved in type I *T*. *cruzi* killing [54]. Still, no systematic study has been conducted that would help us understand the role of ROS in type I parasite proliferation/elimination.

## Heart parasite burden under different redox status

Heart parasite burden may vary after treatment with antioxidants. Assessing the effects of oxidative stress on heart parasite burden is difficult because the published studies are based on different animal models, different stages of infection, and different *T*. *cruzi* strains. Neither BALB/c mice chronically infected with type I Colombian strain and treated with the SOD-mimetic antioxidant tempol [56] nor rats infected with type I Sylvio X10/4 strain and treated since the beginning of infection until the chronic phase with ROS-scavenger phenyl-α-*tert*-butyl (PBN) [23] presented any changes in heart parasite burden. However, BALB/c chronically infected with Colombian strain and treated with resveratrol had reduced heart parasite burden [56]. C57BL/6 mice acutely infected with type II Y strain and treated with CoPP had reduced parasite burden [27], while C3H/HeN mice infected with Sylvio X10/4 and treated with apocynin until chronic stage had increased parasite burden [24]. To explain these results, we should be able to distinguish between different effects of antioxidants at different stages of the disease or different behaviors of type I versus II *T*. *cruzi*.

Recently, Andrade and colleagues showed that the parasite burden of cardiomyocytes infected with type II (JG strain) decreased after treatment with antioxidant enzyme catalase (conjugated to PEG in order to permeate cells), while that of cardiomyocytes infected with type I (Col1.7G2, a clone obtained from Colombian strain), did not [57] (Fig 1H). These results could explain the differences in heart parasite burden after treatment of different infections with antioxidants [25, 27, 56]. Also, resveratrol’s trypanocidal action [58, 59] rather than its antioxidant action may be responsible for the reduction of the parasite burden resveratrol induces at the chronic [56] and acute stages [27].

## The role of oxidative stress in chronic phase cardiomyopathy

Chagas heart disease develops many years after the vector infects the host in about 30%–40% of the patients. A connection between heart disease and infection is still elusive. Most researchers believe Chagas heart disease occurs after cumulative, irreversible tissue destruction. They disagree, however, about the reasons for this tissue damage. Recently, the BENEFIT clinical trial of benznidazole at the chronic stage of Chagas disease has revealed that once established, not even a parasitological cure could prevent the progression of heart disease [60], demonstrating that direct parasite-mediated damage is not responsible for progression.

**Fig 4:**
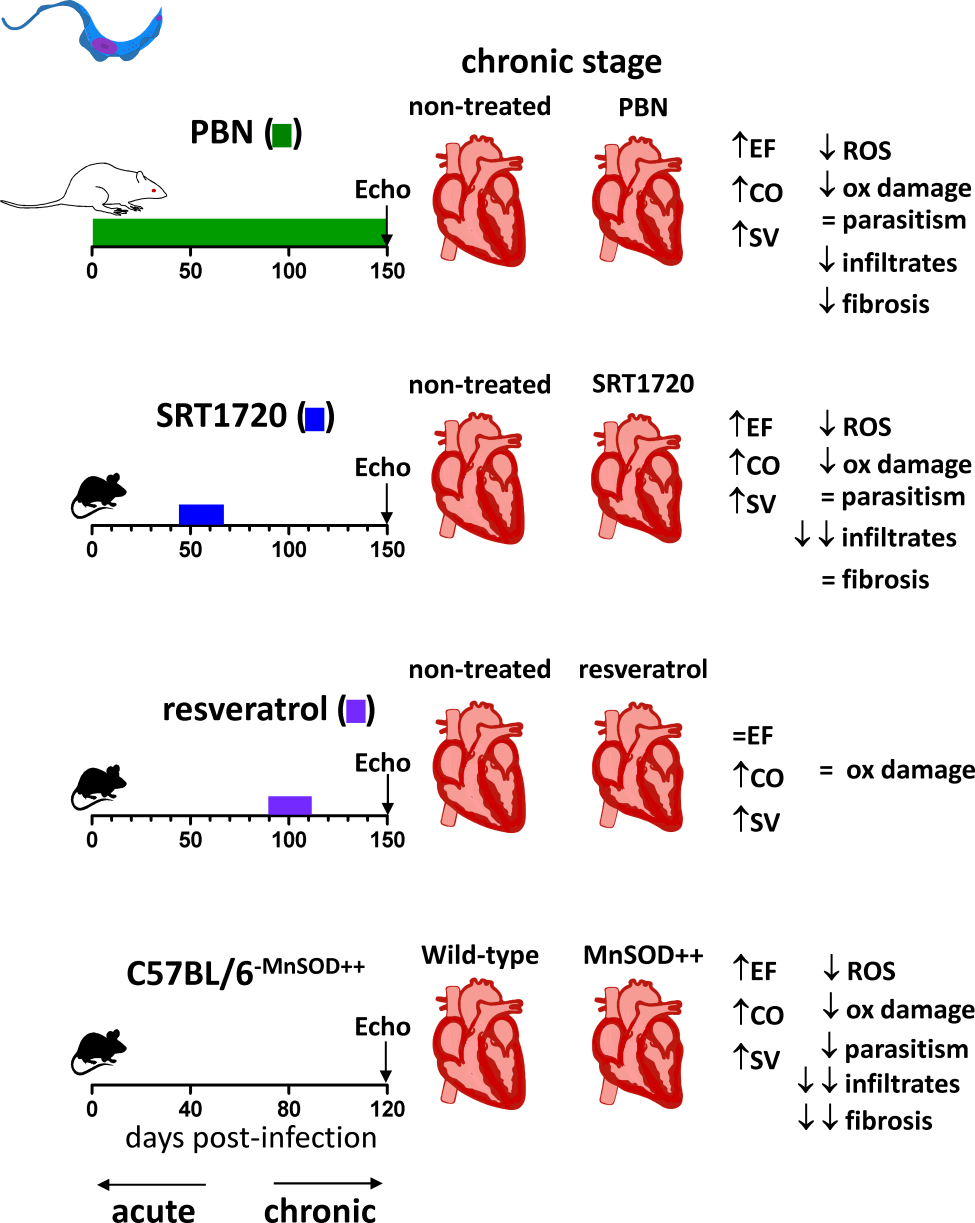
Antioxidants improve cardiac mechanical function in Sylvio X10/4 clone chronically infected animals. Sprague-Dawley rats were infected with Sylvio X10/4 clone and treated with ROS-scavenger PBN from the beginning and throughout infection, improving heart mechanical function [61]. Infected C57BL/6 mice were treated with the antioxidants SRT1720 and resveratrol, resulting in improved heart mechanical function [62]. Infected C57BL/6-MnSOD overexpressing transgenic mice presented healthier mechanical heart function than wild-type mice at the chronic stage of infection [77, 79]. The effects of the antioxidant treatment or genetic modification on ROS, oxidative damage, parasitism, inflammatory infiltrates, and fibrosis are shown for each condition, together with the exact timing of infection and treatment. CO, cardiac output; EF, left ventricle ejection fraction; PBN, phenyl-α-*tert*-butyl; ROS, reactive oxygen species; Mn-SOD, manganese superoxide dismutase.

**Fig 5:**
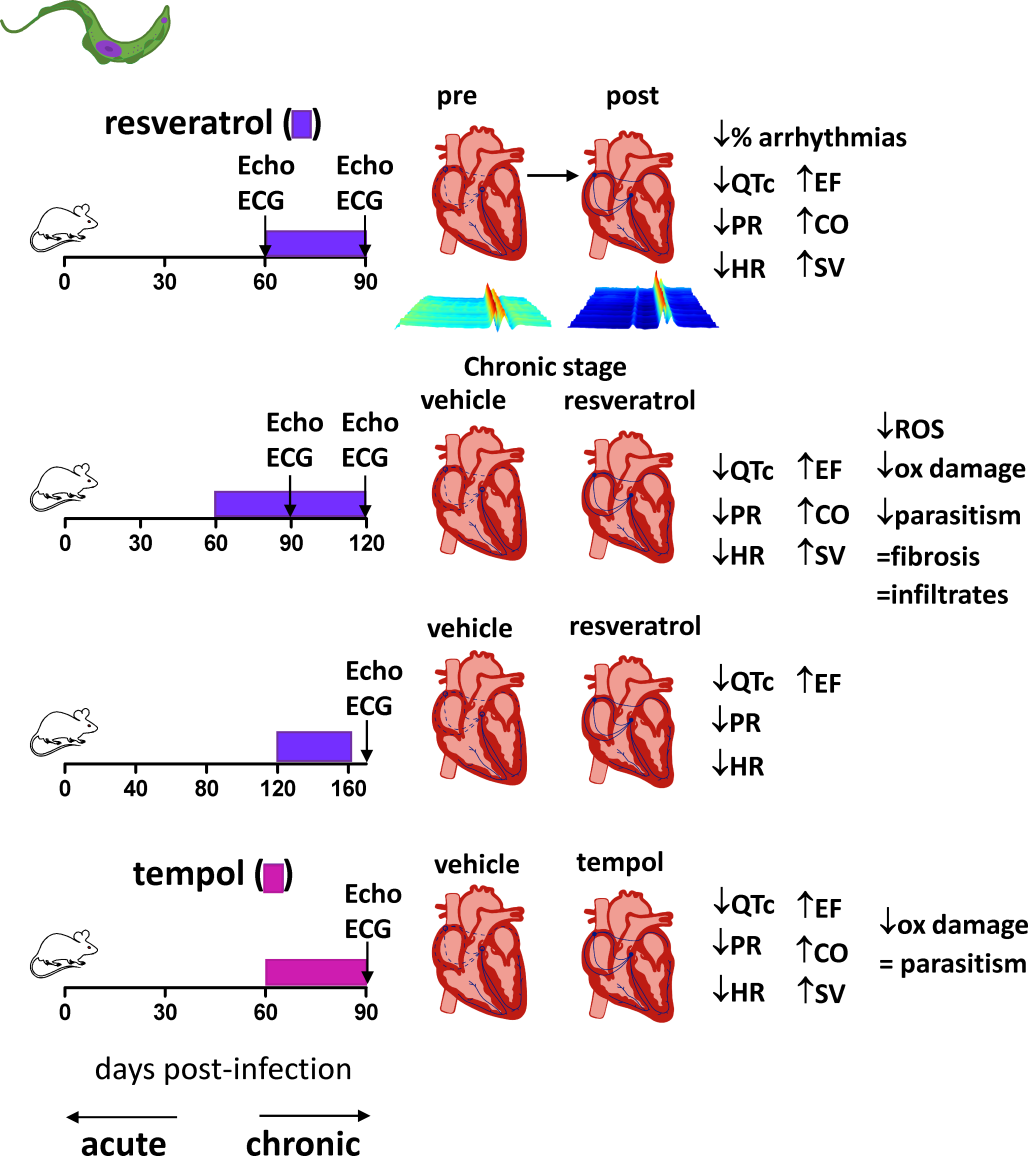
Antioxidants improve cardiac mechanical and electrical function in Colombian strain chronically infected mice. BALB/c mice were infected with *T*. *cruzi* Colombian strain; their heart function was analyzed individually by electro and echocardiography [56]. The mice were treated with antioxidant resveratrol for a month after cardiac disease detection, and the reversal of heart systolic and electrical dysfunction, including arrhythmias, was verified. The treatment with resveratrol or control vehicle was prolonged for an additional month, or performed late after infection, with successful results. The treatment with antioxidant tempol for a month after heart disease detection also improved heart function. The effects of the antioxidant treatment on ROS, oxidative damage, parasitism, inflammatory infiltrates, and fibrosis are shown for each condition, together with the exact timing of infection and treatment. CO, cardiac output; EF, left ventricle ejection fraction; ROS, reactive oxygen species.

A series of works succeeded in preventing [61, 62] and even reversing [56] established functional heart disease with antioxidant therapy in mice, independently of the therapy’s effects on heart parasite burden. These results suggest that Chagas cardiomyopathy is, at least partially, a ROS-dependent pathology [63] (Figs 4 and 5). In cardiomyocytes, ROS elicits production of tumor necrosis factor (TNF) and IL-1β [64], cytokines that may affect heart function [65–67]. ROS may also represent a funnel to which the pathways of several injury-causing factors (such as autoantibodies with adrenergic activities [68–71]) converge to disturb the myocardium physiology, possibly through CAMKII oxidation [69, 72, 73].

Activities of mitochondrial respiratory complexes are depressed in chagasic patients, a phenomenon associated with increased ROS production [74]. Superoxide and H_2_O_2_ are intensively produced in the mitochondria of mice chronically infected with Sylvio X10/4, a process resulting from electron leakage at the respiratory chain [75]. Conversely, treatment with the ROS-scavenger antioxidant PBN improved respiratory chain function and reduced electron leakage in the myocardium of infected mice [76]. In fact, mitochondrial oxidative metabolism was preserved in the hearts of chronically infected mitochondrial SOD (SOD2) superexpressing mice [77]. Altogether, these data indicate that ROS production and mitochondrial dysfunction intertwine in a positive feedback loop in Chagas disease.

Because of oxidative stress, oxidative adducts are found in the heart tissues of both the experimental animals and the chagasic patients [23, 62, 78]. The genetic superexpression of the antioxidant SOD2 preserves mitochondrial structure, abrogates oxidative damage, and inhibits fibrosis and the infiltration of mononuclear cells into heart tissue in the Sylvio X10/4 model [79]. These processes demonstrate that mitochondrial ROS are involved in several of the pathological events of heart disease.

Acute *T*. *cruzi* infection of mice with Sylvio X10/4 clone gave rise to oxidized patterns in myocardium, which could be prevented by treatment with NOX-inhibitor apocynin since day 0 of infection [24] (Fig 4), but the treatment actually increased parasite burden at the acute phase. When the treatment was extended to the chronic phase, no increase was observed in heart weight, cardiomyocyte size, hypertrophy markers, lipid content, and fibrosis. Based on these results, the authors proposed that NOX-derived ROS are responsible for inflammation/cardiac remodeling and for the control of parasite burden in Chagas disease. On the other hand, treatment of infected rats with ROS-scavenger PBN since day 0 of infection did not interfere with chronic parasite burden and only slightly decreased inflammatory infiltrates, but it prevented the formation of oxidized molecular patterns, fibrosis, hypertrophy, and the loss of left-ventricle function [23] (Fig 4). The different effects of apocynin and PBN might result from the different sources of ROS these two antioxidants inhibit, an inconclusive claim for the simple reason that one study used mice [24] while the other used rats [23].

The loss of antioxidant defenses during Chagas disease might underlie the increased oxidative stress. Alternatively, the exhausting oxidative stress during Chagas disease might produce the loss of antioxidant enzymes. Selenium is a cofactor of the antioxidant enzymes glutathione peroxidase (GPX) and thioredoxin reductase (TrxR). Selenium supplementation to mice chronically infected with Brazil strain prevented the prolongation of ECG P wave and the cardiac dilation of the right chamber [80]. Nrf2 is a transcriptional factor that controls gene expression of antioxidant enzymes under oxidative conditions [81]. The declines in left-ventricle function and in the expression of Nrf2, HO-1, and gamma-glutamylcysteine synthetase(GCS) were prevented in the hearts of SOD2 (MnSOD) superexpressing transgenic mice [77] (Fig 4). These data indicate that mitochondrial ROS can both inhibit Nrf2-dependent antioxidant defenses in Chagas disease and impair ventricular function.

The sirtuin 1 (SIRT1) pathway is activated in response to a variety of stressors, such as fasting, and activates a myriad of cell functions by deacetylating key proteins. SIRT1 is connected to the energy-sensing adenosine monophosphate-activated protein kinase (AMPK) pathway. The two pathways seem to activate each other, after which they act together to activate key proteins, such as peroxisome proliferator-activated receptor gamma coactivator 1-alpha (PGC1α) [82]. PGC1α is a transcriptional coactivator that regulates mitochondrial biogenesis, energy expenditure, oxidative phosphorylation (OXPHOS), and antioxidant defenses [83]. The activation of SIRT1 and AMPK pathways has many cardioprotective capacities [84, 85] in ROS-dependent cardiomyopathies, such as diabetes.

Garg and colleagues considered the activation of SIRT1 a potential means to restore mitochondrial respiratory chain activity and OXPHOS capacity as well as to induce mitochondrial biogenesis, processes they found earlier [61, 62] to be impaired in the myocardium of chronically infected rodents. They treated mice chronically infected with type I Sylvio X10/4 clone during the late acute stage with SIRT1 agonist SRT1720 and assessed heart mechanical function during chronic infection [62] (Fig 4). During the infection, SIRT1 activity was depressed in the heart, and SIRT1 agonist treatment improved its activity along with left-ventricular function, although it did not significantly affect cardiac remodeling or interstitial fibrosis. Despite increasing acetylation of PGC1α, the treatment did not improve compromised mitochondrial biogenesis or the expression of mitochondrial DNA–encoded proteins. The authors credited the improvement in cardiac function to the decreased ROS production, oxidative damage, and increased Nrf2 expression they found in the hearts of SRT1720-treated mice; these results indicate that chronic chagasic cardiomyopathy is a ROS-dependent pathology. What must be emphasized, however, is that the authors intervened during the disease development, achieving the long-term prevention, and not reversion, of the disease.

Resveratrol is a multitarget drug with cardioprotective properties exerted through Nrf2, AMPK, SIRT1, SIRT3, and PGC1α [85]. As an attempt to induce tolerance to heart disease, we infected highly susceptible BALB/c mice with the DTU type I Colombian strain and treated them with resveratrol after the disease was established [56]. Treatment reversed individual electrocardiography abnormalities and improved left-ventricular function. The effects of resveratrol could be maintained for long periods of continuous treatment and could be achieved, though smaller, even late after the disease was established. No significant changes in inflammatory infiltrates or interstitial fibrosis were observed in heart tissues. The treatment activated AMPK phosphorylation and increased the expression of mitochondrial SOD2 (MnSOD), in that way reducing heart extravascular ROS, but it did not interfere with SIRT1 expression/activity. Accordingly, treatment with AMPK-activator metformin or with SOD-mimetic tempol were able to mimic resveratrol’s effects on heart function. These results point to a physiological heart dysfunction in Chagas disease, which can be reversed with the appropriate antioxidants and AMPK activators, thus opening a new avenue for the development of specific drugs.

Garg and colleagues also administered resveratrol to mice infected with Sylvio X10/4 [62] (Fig 4). The treatment improved cardiac output, stroke volume, and end systolic volume but did not improve left-ventricle ejection fraction. The difference between two studies were likely due to the different dosages and timing of intervention [56]. We found some improvement in oxidative damage at the chronic stage, but Garg and colleagues did not [56]. Since resveratrol’s half-life is very short, and its effects are usually transient, it is likely that the combination of late intervention, late function assessment, and the low oral dose they used impaired the observation of pronounced, long-lasting effects.

The association between parasite burden and functional chronic heart disease has been challenged by many studies [60]. In fact, in murine experimental Chagas disease, the prevention/reversion of cardiac dysfunction was accompanied by unchanged [56], increased [86], or decreased parasite burden [56]. Therefore, varying results corroborate the idea that at the chronic stage of infection, parasite burden is no longer responsible for heart dysfunction. Also, the association between inflammatory infiltrates and cardiac dysfunction in Chagas heart disease is far looser than one might expect [87]. Treatments successful in preventing/reversing cardiac dysfunction have been found to either reduce [86] or not interfere [23, 56] with heart inflammatory infiltrates and fibrosis in murine experimental Chagas disease.

The evidence presented in this session suggests oxidative stress is an important pathogenic factor in Chagas cardiomyopathy. The inhibition of oxidative stress seems to be critical to both improving and preventing heart dysfunction from developing. The interference with parasite burden, interstitial fibrosis, inflammatory infiltrates, and cardiac hypertrophy may either accompany functional improvement or not [56, 62, 80, 86]. A first antioxidant tested as a therapy to Chagas disease was vitamin E. It reversed oxidative damage [88] and, along with benznidazole, prevented oxidative damage and reduced the incidence of premature ventricular contraction (PVC) [89]. Selenium is being tried as a therapy [90], and we expect that soon indirect antioxidants that act through Nrf2 or SIRT1 pathways should find their way to clinical trials.

## Outstanding questions for further scrutiny

The above analysis of the subject literature led us to formulate the following challenging questions to answer:

- How is it possible that, unlike other microorganisms, *T*. *cruz*i has evolved to thrive in oxidative conditions?
- Can preconditioning against oxidative stress prevent the initial spread of infection?
- Do all ROS work in the same way? Does *T*. *cruzi* grow in response to ROS in all cell types?
- Do ROS act directly on the parasite by signaling to enhance its growth, and/or do they act by transforming the environment in which amastigotes grow?
- Do *T*. *cruzi* from different DTUs behave differently in response to ROS? Are conflicting results in the literature due to different strains used?
- What can explain the unchanged or even increased parasite burden found after treatment with antioxidants at the chronic stage, unlike treatment applied at the acute stage?
- Can we inhibit ROS production without interfering with *T*. *cruzi* elimination by activated macrophages? Is peroxynitrite indispensable to the killing of *T*. *cruzi* by activated macrophages in vivo, or does NO alone suffice to kill it?
- Are ROS involved in both physiological, reversible heart dysfunction and cumulative, progressively destructive cardiomyopathy?
- Can antioxidant therapy prevent the progression of human Chagas heart disease or even reverse it?

Such knowledge could help reconcile conflicting literature on the role of ROS in *T*. *cruzi* infection, potentially helping develop new therapies.

## Concluding remarks

The recent literature has pointed toward oxidative stress as a villain in Chagas disease: It helps parasites to grow during the acute stage of infection, and it participates in the generation of cardiomyopathy at the chronic stage. Although the reduction of parasite burden seems useless at the chronic stage, a plethora of data suggest antioxidant therapy as a candidate treatment to chronic Chagas disease cardiomyopathy. We still need to learn a lot about the effects of antioxidant therapy on infection by different *T*. *cruzi* strains. Crucial knowledge could be gained by research that would tightly control parasite burden, thanks to which therapy’s efficiency could be tuned up and patients’ safety could be ensured. Even despite these gaps in the knowledge, however, antioxidants are—for the moment—the most promising road to a specific treatment in Chagas disease.
